# Ultrastructural mapping of salivary gland innervation in the tick *Ixodes ricinus*

**DOI:** 10.1038/s41598-019-43284-6

**Published:** 2019-05-02

**Authors:** Marie Vancová, Tomáš Bílý, Jana Nebesářová, Libor Grubhoffer, Sarah Bonnet, Yoonseong Park, Ladislav Šimo

**Affiliations:** 1 Laboratory of EM, Institute of Parasitology, Biology Centre of CAS, České Budějovice, Czech Republic; 2 Faculty of Science, University of South Bohemia, České Budějovice, Czech Republic; 3 Faculty of Science, Charles University in Prague, Prague, Czech Republic; 4 UMR BIPAR, INRA, Ecole Nationale Vétérinaire d’Alfort, ANSES, Université Paris-Est, Maisons-Alfort, France; 5 Department of Entomology, Kansas State University, 123 Waters Hall, Manhattan, KS 66506 USA

**Keywords:** Neuroscience, Cryoelectron microscopy

## Abstract

The salivary gland of hard ticks is a highly innervated tissue where multiple intertwined axonal projections enter each individual acini. In the present study, we investigated the ultrastructural architecture of axonal projections within granular salivary gland type II and III acini of *Ixodes ricinus* female. Using immunogold labeling, we specifically examined the associations of SIFamide neuropeptide, SIFamide receptor (SIFa_R), neuropeptide pigment dispersing factor (PDF), and the invertebrate-specific D1-like dopamine receptor (InvD1L), with acinar cells. In both acini types, SIFamide-positive axons were found to be in direct contact with either basal epithelial cells or a single adlumenal myoepithelial cell in close proximity to the either the acinar duct or its valve, respectively. Accordingly, SIFa_R staining correlated with SIFamide-positive axons in both basal epithelial and myoepithelial cells. Immunoreactivity for both InvD1L and PDF (type II acini exclusively) revealed positive axons radiating along the acinar lumen. These axons were primarily enclosed by the adlumenal myoepithelial cell plasma membrane and interstitial projections of ablumenal epithelial cells. Our study has revealed the detailed ultrastructure of *I. ricinus* salivary glands, and provides a solid baseline for a comprehensive understanding of the cell-axon interactions and their functions in this essential tick organ.

## Introduction

**Figure 1:**
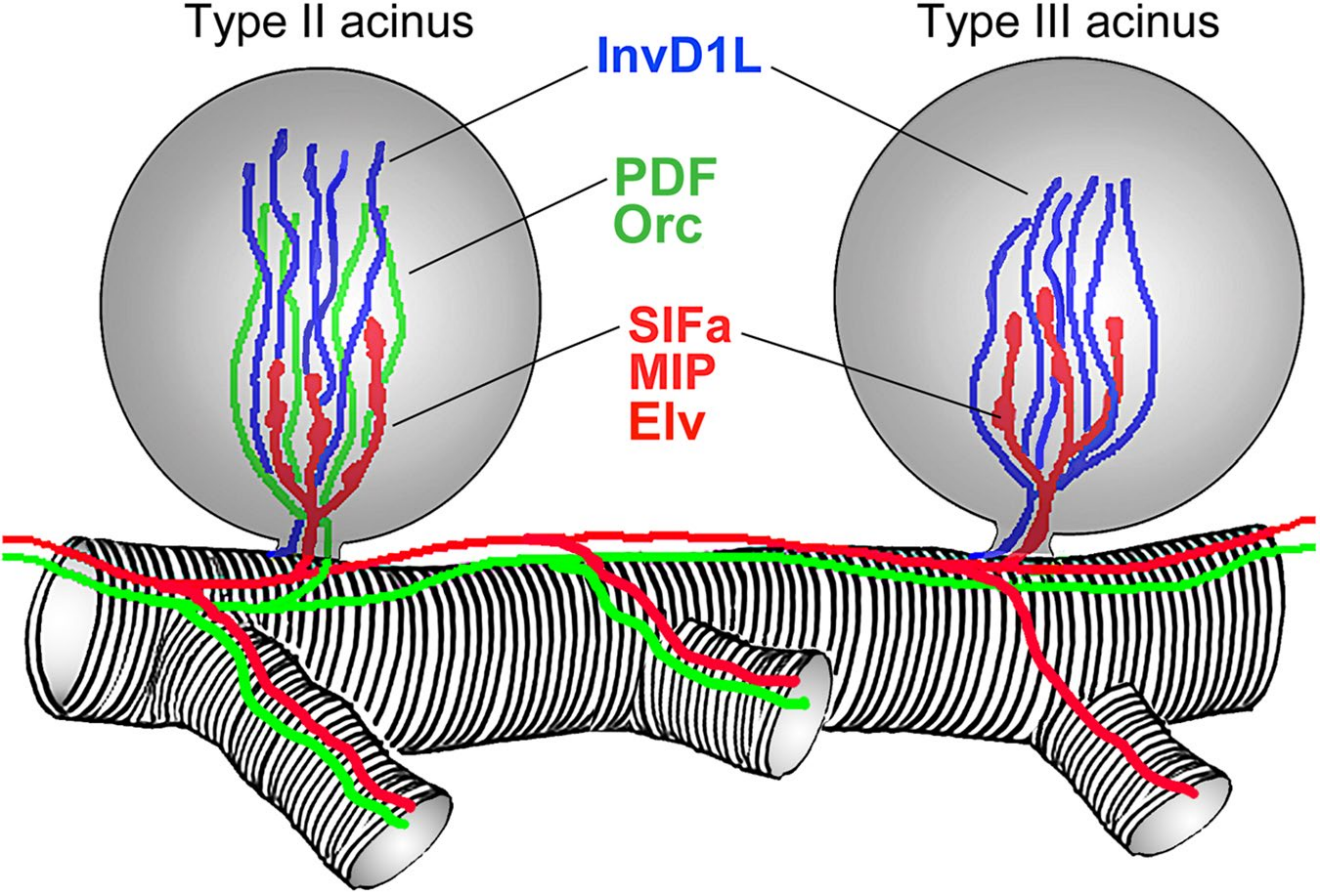
Schematic drawing of type II and III acini innervations in salivary glands of unfed *Ixodes scapularis* females. Note that three and two different innervations were found in type II and III acini respectively. SIFa/MIP/Elv- (red) and PDF/Orc-positive (green) axons arise from specific neurons in tick synganglia^17,18,21,22^, while the source of InvD1L axons (blue) remain obscured^19^. Note that in the wholemount immunohistochemistry the SIFa/MIP/Elv-axons also cross-react with the InvD1L antibody^19^ (not shown in the schema), which has not been confirmed in current study. InvD1L: invertebrate-specific D1-like dopamine receptor; PDF: pigment dispersing factor; Orc: orcokinin; MIP: myoinhibitory peptide; SIFa: SIFamide; Elv: elevenin.

Tick salivary gland (SG) secretions eliminate excess water and ions from tick bodies to maintain homeostasis^1^ and simultaneously compromise the host’s defense system to ensure long-term feeding success^2,3^. The SG of hard female ticks is composed of three distinct types of acini that differ both morphologically and functionally^4,5^. The agranular type I acini are known to aid tick homeostatic maintenance during both on- and off-host periods by absorbing atmospheric water and ions^6,7^, while granular type II and III SG acini share similar structural characteristics, and form highly complex secretory units that secret either biologically active molecules or fluids^8–10^. The complex acinar cell configuration^11–13^ required to secrete the various different substances^2^ is what characterizes the precise and dynamic control mechanisms of this highly versatile organ. Neural control of the tick SG has been extensively studied in various tick species^14–17^, and the most detailed information to date has been demonstrated in the North American black-legged tick *Ixodes scapularis*^18–21^. Previously, using specific antibodies, numerous axonal processes arborizing within type II and III acini have been identified (Fig. 1): (i) neuropeptidergic axon terminals co-expressing myoinhibitory peptide (MIP), SIFamide and elevenin (Elv) terminate at the basal regions in both type II and III acini^18,21^; (ii) neuropeptidergic axon terminals positively immunoreacting to antibodies against both pigment dispersing factor (PDF) and orcokinin only reach the apical parts of type II acini^17,22^; and (iii) axonal projections expressing invertebrate-specific D1-like dopamine receptor (InvD1L) reaching the apical regions of both type II and III acini were identifed^19^. Surprisingly, the InvD1L dopamine receptor antibody also recognized basal MIP/SIFamide/Elv axons in both type II and III acini in a previous study^19^. In addition, SIFamide receptor (SIFa_R) was detected in the basal region of both type II and III acini, in close association with the SIFamide-releasing axon terminals (Fig. 1)^20^.

Recent *in vitro* physiological experiments demonstrated that dopamine-activated expulsions of acinar content to the adjacent ducts is mediated via the contractile myoepithelial cell (MC)^23^. The single adlumenal MC lines the entire acinar lumen—including the region above the acinar valve—in a web-like fashion^13,19,24^. Combining both morphological and physiological data, we have suggested that the MC is a target for downstream signals of InvD1L-positive axons, whereas basally located MIP/SIFamide/Elv-releasing axon terminals may play a role in the modulation of paracrine/autocrine dopamine actions in the acini and/or control the acinar valve via the MC^19,20,25^. Although it appears that the MC is controlled by different axonal projections^19,20,25^, detailed knowledge about the specific cell(s)-axon(s) connections remains enigmatic, mainly due to difficulties in distinguishing clear acinar cell boundaries via wholemount immunohistochemistry (IHC) approaches.

Therefore, we used immunogold labeling and transmission electron microscopy (TEM) to investigate SIFamide, SIFa_R, InvD1L, and PDF, to further our understanding of type II and III SG acini innervations in unfed *Ixodes ricinus* females. Taking advantage of the high contrast TEM image generated by *en bloc* hafnium chloride staining during the freeze substitution technique^26,27^ we were able to trace the borders of distinct acinar cells near the acinar duct and valve and recognize associated axons with this highly complex region. The 3D reconstructions of double-immunogold-stained structures identified distinct innervations, thus revealing their relationship with specific acinar cells for the first time.

## Material and Methods

### Ticks

Adult *I. ricinus* females were obtained from the tick rearing facility at the Institute of Parasitology of the Biology Centre, Czech Academy of Sciences, Czech Republic. Ticks were maintained in plastic vials containing sterile wood shavings and covered by cotton at >90% relative humidity and 22 °C. All ticks used in the study were unfed *I. ricinus* females.

### High pressure freezing and freeze substitution

For ultrastructural studies, SG of unfed *I. ricinus* females were dissected in 0.1 M HEPES and immediately frozen using the Leica EM PACT high pressure freezing (HPF) system in the presence of 20% bovine serum albumin (Sigma-Aldrich). Freeze substitution was performed in 2% OsO_4_ (EMS) in 100% acetone (−90 °C, 96 h). Then temperature was increased to −60 °C (1.9 °C/1 h) and after 24 h up to −30 °C (1.3 °C/h). At −30 °C, OsO_4_ was replaced with 1% thiocarbohydrazide (Sigma); then with 2% OsO_4_; and finally with either 1% uranyl acetate (EMS) or 1% HfCl_4_ (Sigma). All solutions were diluted in 100% acetone with a 24 h incubation period at each step. After increasing the temperature to 4 °C (1.4 °C/h) the samples were washed three times for 15 minutes in 100% acetone, infiltrated and embedded in EMBed 812 resin (EMS). Ultrathin sections were subsequently counterstained in ethanolic uranyl acetate for 30 minutes and lead citrate for 20 minutes.

### Antibodies and immunoreactive epitopes

The antibodies used in this study are listed in Table 1. The neuropeptide antibodies were well characterized in wholemount immunohistochemistry of various tick species^16,21,22^. The InvD1L and SIFa-R receptor antibodies were raised against the last 20 amino acids in the C-terminal *I. scapularis* protein sequence^19,20^. While the C-terminal sequences of the G-protein coupled receptors often diverge among the different arthropod taxa, we examined whether *I. ricinus* and *I. scapularis* share the same SIFa_R and InvD1L C-terminal protein sequences. SIFa_R and InvD1L selected regions were amplified from cDNA obtained from the SG of partially-fed *I. ricinus* females. PCR products were cloned into the pGMTeasy vector (Promega) and sequenced. For the sequence analyses, see Supplementary Fig. S1.

**Table 1:** List of antibodies used in this study.

Antibody name	Host	Source/characterization	Dilution for TEM	Immunogen	Secondary probe conjugated to gold nanoparticles (nm)
Drome SIFamide	Rabbit polyclonal	^35^	1:30	AYRKPPFNGSIFamide	protein A (5) or goat α-rabbit IgG (15 nm)
Ucapu PDH*	Rabbit polyclonal	^36^	1:20	NSELINSILGLPKVMNDAamide	goat α-rabbit IgG (6 nm or 15 nm)
Ixosc SIFa_R	Chicken polyclonal, affinity purified	^20^	1:20	CTRGLSRYDTQCEYLSTSAV	goat α-chicken IgY (6 nm)
Ixosc InvD1L	Chicken polyclonal, affinity purified	^19^	1:30	CRIIKEDASMRSQSLEEAVL	goat α-chicken IgY (6 nm or 15 nm)

^*^The antibody against *Uca pugilator* pigment dispersing hormone (PDH)^36^ has been shown to recognize various neurons in *Drosophila melanogaster*^37^ and in different tick species^16,17,22^. Because there is no evidence about hormonal actions of PDH in insect neither in ticks we use the term pigment dispersing factor (PDF) for ours immunoreactivities with this antibody. GenBank accession numbers for: *D. melanogaster* SIFamide, NM_001259567; *U. pugilator* PDH, 1109281A; *I. scapularis* SIFa_R, AGE11606; *I. scapularis* InvD1L, XM_002399612.1.

### Immunogold labeling of thawed cryosections (Tokuyasu technique)

Dissected SGs were fixed in a mixture of 4% formaldehyde + 0.1% glutaraldehyde in 0.1 M HEPES (Sigma-Aldrich) for 1 h at room temperature. After washing in HEPES containing 0.02 M glycine, the tissues were embedded into 10% gelatin (Serva Electrophoresis) and cryoprotected in 2.1 M sucrose solution for 24–72 h at 4 °C and then frozen in liquid nitrogen. Ultrathin cryosections were cut using the cryo-ultramicrotome LEICA EM UC-6/FC-6 at temperatures ranging from −120 °C to −100 °C. The sections were picked up using a drop of 1% methyl cellulose/1.05 M sucrose mixture^28^ and transferred onto Formvar-carbon coated grids. The sections were treated with blocking buffer (BB) composed of 1% fish skin gelatin and 0.05% Tween 20 (Sigma-Aldrich) dissolved in HEPES, and then incubated in antibodies diluted in BB (Table 1) for 1–2 h. After washing in BB, the sections were incubated with various secondary probes conjugated to different sized gold nanoparticles (Table 1): e.g. protein A, 5 nm (UMC, Utrecht); goat anti-rabbit IgG conjugated either to 6 nm (BBI) or 15 nm (Aurion); and goat anti-chicken IgY, 6 nm or 15 nm (Aurion) particles. Conjugates were diluted 1:50 in BB and incubated with samples for 1 hour at room temperature. Controls for nonspecific binding of the secondary antibody were performed by omitting the primary antibody. Finally, sections were washed in HEPES, dH_2_O, and then contrasted/embedded using a mixture of 2% methylcellulose and 3% aq. uranyl acetate solution (9:1). Samples were observed using a JEOL 1010 TEM or a JEOL 2100-F TEM equipped with a high-tilt stage and a Gatan camera (Orius SC 1000), controlled with SerialEM automated acquisition software. Tilt series images were collected in the range of ±60° to 65°, with 0.6° to 1° increments. Electron tomograms were reconstructed with the IMOD software package. Manual masking of the area of interest was employed to generate 3D models. 3D model visualizations were performed in IMOD and Amira (ThermoFisher Scientific) software packages respectively. Coloring of cell types/structures from TEM images was performed in Adobe Photoshop.

## Results

### Ultrastructure of acinar cells and associated axonal projections

**Figure 2:**
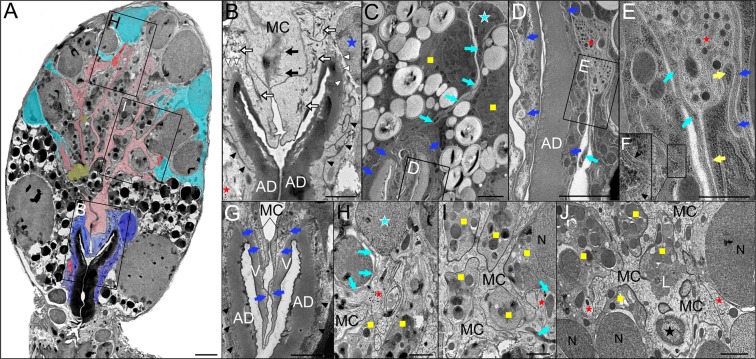
Transmission electron microscopy image of type III acinus from unfed *Ixodes ricinus* female salivary glands highlighting the cellular composition and associated axons. (**A**) Cross-section through the middle of the acinus reveals common axon positions (red), basal epithelial cells (ECs, dark blue), myoepithelial cell (MC, pink), ablumenal epithelial cells (AECs, aqua-blue) and lumen (yellow). Different granular cells at both basal and apical regions of the acinus are left in black and white. Insets in A are magnified in (**B,H,I**). (**B**) The acinar valve region. Apparent microtubules (black arrows) in the MC and (white empty arrows) in ECs are shown. EC labyrinthine junctions (black arrowheads) are closely associated with the acinar duct (AD). Basal canaliculi (white arrowheads) of ECs are evident on the AD apex. The blue asterisk shows the nucleus of the EC. The basal axon is indicated by the red asterisk. (**C–E**) The long AEC (aqua-blue asterisk) projections (aqua-blue arrows) between two granular cells (yellow squares) reaching the surrounding area of the AD. Inset in (**C**) is magnified in (**D**,**E**). (**D–E**) A prominent axon (red asterisk) containing neurosecretory vesicles is enclosed by ECs (blue arrows), AEC projections (aqua-blue arrows), and a granular cell (yellow arrows in E). Note that axons are never in direct contact with the AD. (**F**) Septate junctions (black arrowheads) present at the site of interdigitation between neighboring ECs. (**G**) The acinar valve (V) is covered by two layers of cells, the ECs (blue arrows) and the MC. Labyrinthine junctions (black arrowheads) of the ECs closely surround the AD. (**H–J**) The axons (red asterisks) are in direct contact with the MC’s finger-like projections which radiate between granular cells (yellow squares) and fine projections of AECs (aqua-blue arrows in **H**,**I**). Aqua-blue asterisk in H shows the nucleus of AEC. The MC nucleus (black asterisk) is shown in (**J**). N: nucleus; L: lumen. Specimens were prepared by HPF/FS and a modified OTO method with *en bloc* hafnium chloride (**A,B,H–J**) or uranyl acetate (**C–G**) staining. Scale bars: 5 μm (**A**), 2 μm (**B–D,G–J**), 500 nm (**E**).

The spatial organization of acinar cells, including associated axons containing neurosecretory vesicles, is revealed with low-magnification TEM imaging of a type III acinus (Fig. 2A). The basally located epithelial cells (ECs), tightly surround the entire chitinous acinar duct and also overlay the arms of the acinar valve (Fig. 2A–G). These cells possess a relatively large nuclei close to the apex of the acinar duct and abundant bundles of microtubules are evident at valve-adjoining regions (Fig. 2A,B). Highly labyrinthine EC plasma membranes form numerous septate junctions, which are primarily evident on the sides of the acinar duct (Fig. 2B,F,G). Basal canaliculi formed by EC membranes are mainly apparent at the apex of the acinar duct (Fig. 2B). Multiple axons containing neurosecretory vesicles were found adjacent to the acinar duct. In very basal region of the acini, axons were exclusively encapsulated by convoluted EC plasma membranes (Fig. 2A,B), effectively separating axons from direct contact with chitinous acinar ducts. Axons—running at or around the level of the acinar valve—were enclosed between interdigitated EC plasma membranes and interstitial extensions of ablumenal epithelial cells (AECs), which extend between the granular cells towards the acinar duct (Fig. 2C–E). Occasionally, these axons made contact with large basal granular cells (Fig. 2E).

A single ablumenal MC lined the entire acinar lumen, and its finger-like projections radiated between the individual granular cells towards the basement membrane, where they formed intimate connections with AEC terminal extensions (Fig. 2A,H–J). The nucleus of this cell, which is often difficult to locate in TEM sections, was recognized at approximately one-third length of the acinus closest to the lumen (Fig. 2J). The basal regions of the MC overlay approximately half the length of the EC extensions directly covering the acinar valve (Fig. 2A,B,G). Copious microvilli were detected on the MC’s lumenal side (Fig. 2J). The microtubule bundles were identified across the entire body of the MC, and were more densely packed at contact zones between basal parts of the MC in the region above the acinar valve (Fig. 2B). Scattered axonal projections enclosed by MC and AEC plasma membranes were frequently detected (Fig. 2A,H–J), but axons possessing direct contact with granular cells (type F) were less common (Fig. 2H).

**Figure 3:**
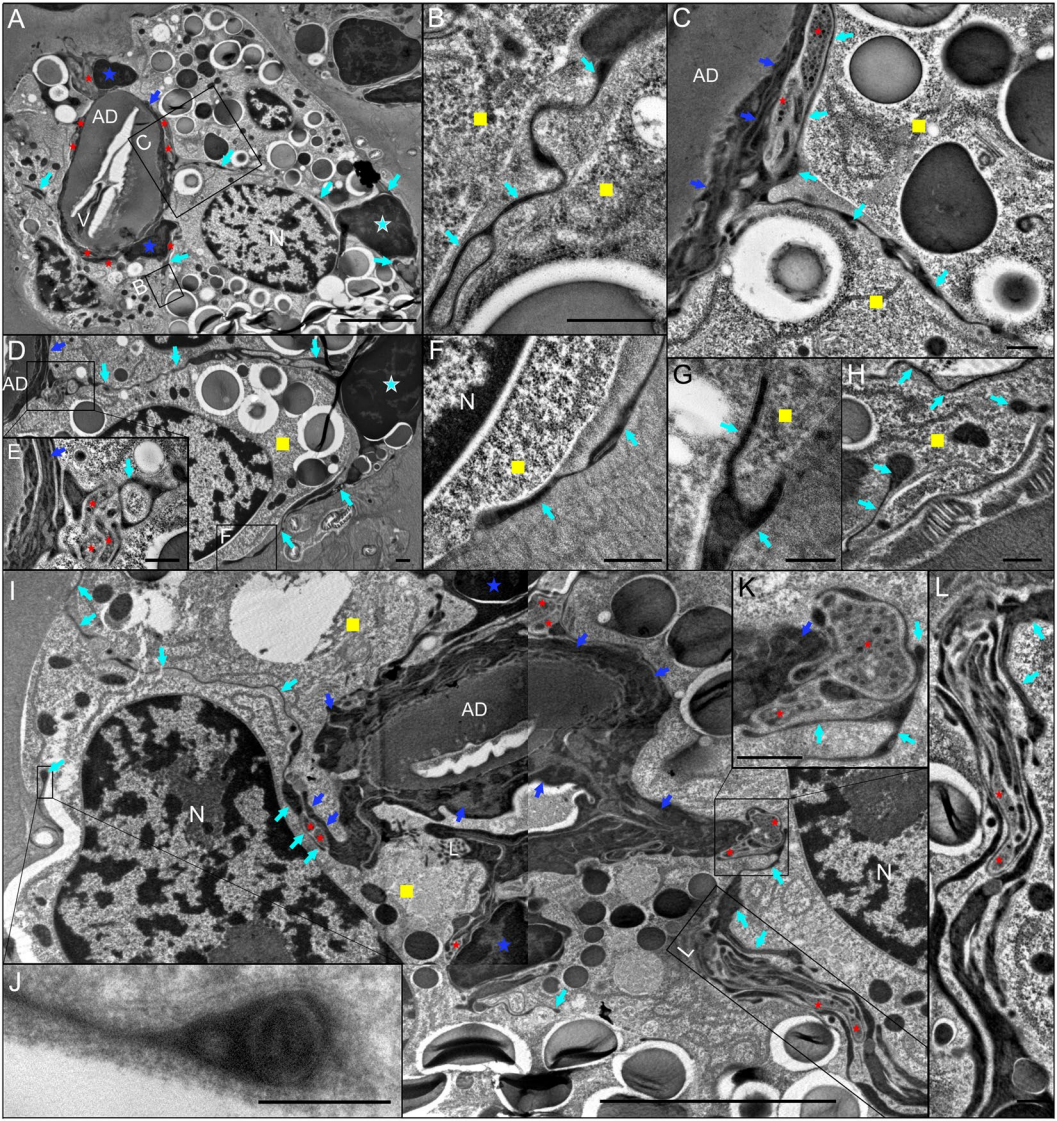
Transmission electron microscopy image of the region surrounding the acinar duct in type II acinus of unfed female *Ixodes ricinus* salivary glands. (**A–C**) Multiple axonal projections (red asterisks) run closely along the acinar duct (AD) and are enclosed by basal epithelial cells (ECs, blue arrows) and the extensions of ablumenal epithelial cells (AEC, aqua-blue arrows). Nuclei of ECs (blue asterisks in A) and of AEC (aqua-blue asterisk in A). Insets in A are magnified in (**B,C**). For 3D animation of panel (A) see Fig. 4 and Video 1. (**B**) Detailed view of AEC extension (aqua-blue arrows) surrounded by extracellular matrix and radiating between two granular cells (yellow squares). (**C**) Detail of axons surrounded by the ECs (blue arrows) and AEC projection (aqua-blue arrows). (**D,E**) Different depth section of the region shown in (**A**). The interstitial projections (aqua-blue arrows) of AEC, located under the basal lamina (nucleus of AEC is labeled with aqua-blue asterisk), reach the region of the acinar duct, and along with ECs (blue arrows) enclose the axons (red asterisks). Inset in (**D**) is magnified in (**F**). (**F–H**) Under the acinar basement membrane, different AEC projections (aqua-blue arrows) invaginate granular cells (yellow squares). (**I–L**) Further in-depth section of the same acinus highlighting the associations of the basal axons (red asterisk) with the ECs (blue arrows) and interstitial AEC projections (aqua-blue arrows). Blue asterisks show the nuclei of ECs and yellow squares indicate granular cells. (**J**) Invagination of AEC projection into the granular cell under the basement membrane. (**K,L**) Detailed views of axons (red asterisks) associated with the ECs (blue arrows) and AEC projections (aqua-blue arrows). L: lumen; V: valve; N: nucleus of granular cell. Specimens were prepared by HPF/FS using OTO and *en bloc* hafnium staining. Scale bars: 5 μm (**A,I**), 500 nm (**B–E,H,K,L**), 200 nm (**G,F,J**).

**Figure 4:**
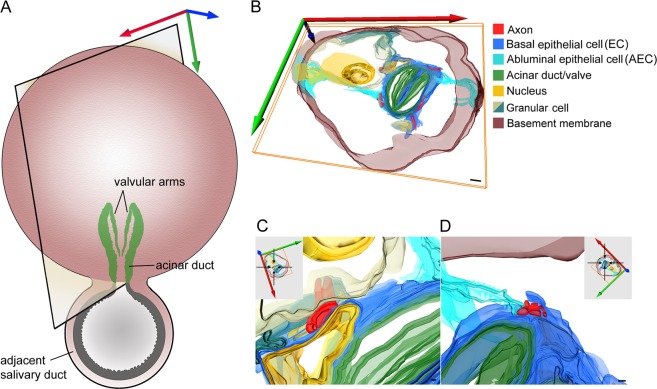
Schematic 3D model of basal region of the type II acinus (Fig. 3A), reconstructed from serial ultrathin sections of unfed *Ixodes ricinus* female salivary glands. (**A**) Schematic image of acinus indicating the position of the reconstructed area highlighted in panel (B). (**B**) Axonal projections (red) running along the acinar duct (green) are encapsulated by basal epithelial cells (ECs, blue) and projections of ablumenal epithelial cells (AECs, aqua-blue). (**C,D**) Magnified regions of image (**B**), with coordinates shown in their insets. Note that ECs separate the axons from direct contact with the acinar duct. Occasional contact of axons with the granular cell (grey/greenish) is detailed in (**C**). For 3D animation see Video 1. Scale bars: 2 µm (**B**), 200 nm (**C,D**).

In type II acini, up to eight axonal projections in close alignment to the acinar duct have been observed (Fig. 3A). AECs extend very fine projections throughout the extracellular matrix between basal granular cells and towards the acinar duct to form intercellular connections between axons and their surrounding ECs (Fig. 3A–L). Near the acinar basement membrane, AEC projections can be seen to invaginate the cytoplasm of granular cells (Fig. 3H–J). A 3D model reconstructed from serial ultrathin resin sections reveals further insights into the spatial organization of the type II acinus basal region, including the different acinar cells encapsulating axons surrounding the acinar duct/valve (Fig. 4, Video 1). Similar to type III acini, axons adjacent to the apex of the acinar duct make direct contact with ECs, terminal extensions of AECs, and basal granular cells. In unfed *I. ricinus* females, the lumen in type II acini is proportionally larger compared to type III. Morphological features of the MC lining the lumen and part of the acinar valve region are shared between these two acini types (see Fig. 2A,B,G–J).

For ultrastructural studies, we used osmium-thiocarbohydrazide-osmium methods followed by *en-bloc* staining with either uranyl acetate (Fig. 2C–G) or hafnium chloride (Figs 2A,B,H–J and 3) to enhance contrast of membranes and microtubules. In comparison to uranyl acetate (Fig. 2G), hafnium staining repeatedly and significantly enhanced the contrast of microtubules (Fig. 2B) and, in several acini, increased the cytoplasm density of both ECs and AECs (Fig. 3).

### SIFamide and SIFamide receptor

**Figure 5:**
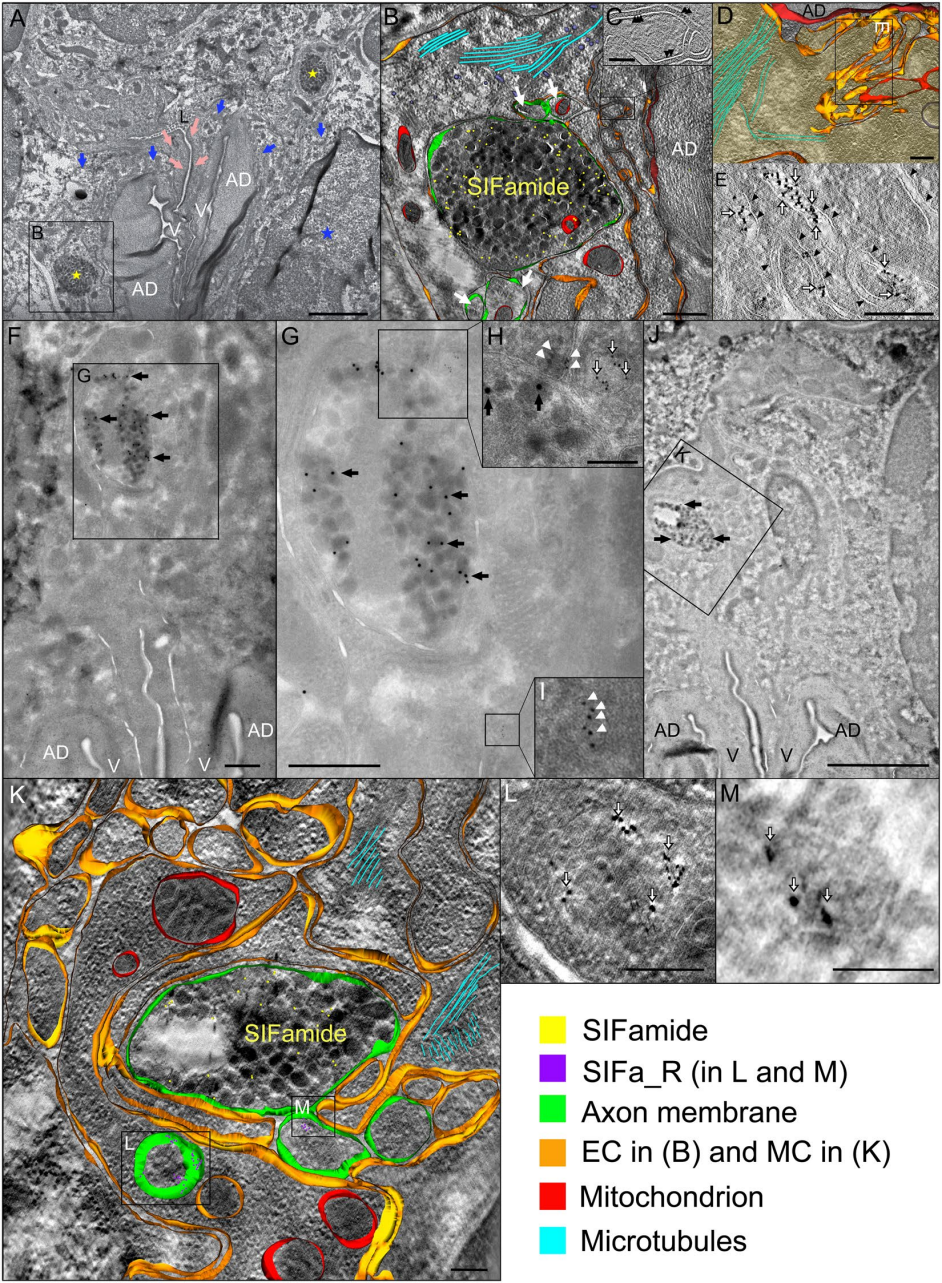
Transmission electron microscopy image showing immunogold labeling of SIFamide and SIFamide receptor (SIFa_R) in type III acinus of unfed *Ixodes ricinus* female salivary glands. (**A**) The SIFa-axons (yellow asterisks) were found in close proximity to the acinar duct (AD) and its valve (V). Myoepithelial cell (MC, pink arrows) and basal epithelial cells (ECs, blue arrows) surround the acinar valve (see also Fig. 2A,B,G). The inset in A is magnified in (**B**). (**B**) Basal SIFa-axon containing electron-dense neurosecretory vesicles (yellow dots indicate the reaction with SIFamide antibody). Four small axons (white arrows) containing electron-lucent vesicles are associated with the large SIFa-axon. Note that the axons (green) are separated from the acinar duct (AD) and are enclosed by highly convoluted ECs (gold) forming septate junctions (black arrowheads in C). Mitochondria (red) within and near the axons, and apparent microtubules (aqua-blue) are shown. For 3D animation of panel (B) see Video 2. (**D**,**E**) SIFa_R staining (white empty arrows in E show 6 nm nanoparticles) is observed on the EC canaliculi in the AD apex region. Black arrowheads in E show EC membranes. (**F–M**) Double labeling of SIFamide (black arrows indicate 15 nm nanoparticles) and SIFa_R (white empty arrows or white arrowheads show 6 nm nanoparticles) in the region above the acinar valve. Insets in F and J are magnified in (**G**) and (**K**) respectively. Insets in K are magnified in (**L,M**). Note that two different SIFa_R reactions were recognized in close proximity to the prominent SIFa-axon (**H,I,K–M**). First, SIFa_R-positive vesicle-like structures (white arrowheads in **H**,**I**) and the MC plasma membrane (white arrowheads in **H**). Second, the small axons containing electron-lucent vesicles accompanying the SIFa-axon were also positive for SIFa_R (white empty arrowheads in **H,L**,**M**). Numerous mitochondria (red) and microtubules (aqua-blue) were recognized in the MC (gold in **K**) surrounding the axons (green). (**B,D,K**) Overlay of one tomographic slice and 3D rendered model, single-axis electron tomography (ET). (**C,E,H,L,M**) Slices of ET. Scale bars: 2 μm (**A,J**), 500 nm (**B,F,G**), 200 nm (**D,E,H,K,L**), 100 nm (**C,M**).

In both type II and III acini, immunogold labeling revealed direct associations of SIFamide and its receptor within both ECs and the MC, in the vicinity of the acinar duct and its valve, respectively (Fig. 5, Supplementary Fig. S2). In type III acini large caliber (~5 μm) SIFamide-positive axons (SIFa-axons) accompanied by four proportionally smaller diameter (~500 nm) SIFamide-negative axons were detected near the acinar duct basal region. SIFa-axons contained electron-dense neurosecretory vesicles, while electron-lucent vesicles were more frequently observed in the smaller accompanying axons (Fig. 5A,B). All identified axons in this region were surrounded by convoluted EC plasma membranes containing microtubule bundles and abundant mitochondria (Video 2). Mitochondria were also observed in SIFa-axons and two of the associated axons (Fig. 5B). In numerous TEM sections, we noted that septate junctions between individual ECs appeared to be more abundant in zones between axons and the acinar duct (Fig. 5B,C). In addition, the EC basal plasma membranes form a labyrinth of canaliculi where ECs directly contact the apical region of valvular arms. Interestingly, these structures were positive for SIFa_R-immunogold labeling in both type of acini (Fig. 5D,E, Supplementary Fig. S2), predominantly at the extracellular membranes as observed with electron tomography (Fig. 5E). Furthermore, the MC (predominantly in acini type III) was also associated with both SIFamide and SIFa_R together (Fig. 5F–M). This co-labeling revealed positive immunoreactions in the region just above the acinar valve, where highly labyrinthine MC plasma membrane encloses large-caliber (~2.5 μm) SIFa-axons, accompanied by three smaller-diameter (~400 nm) axons with electron-lucent vesicles (Fig. 5F–K). In type III acini, positive SIFa_R staining was observed within two of these electron-lucent axons (Fig. 5H,L,M). In the MC cytoplasm, small vesicle-like structures were also SIFa_R positive (Fig. 5I). In addition, MC plasma membranes enclosing the SIFa-axon were positive for SIFa_R staining (Fig. 5H) as revealed by virtual electron tomography Z- slices.

### Invertebrate-specific D1-like dopamine receptor and SIFamide

**Figure 6:**
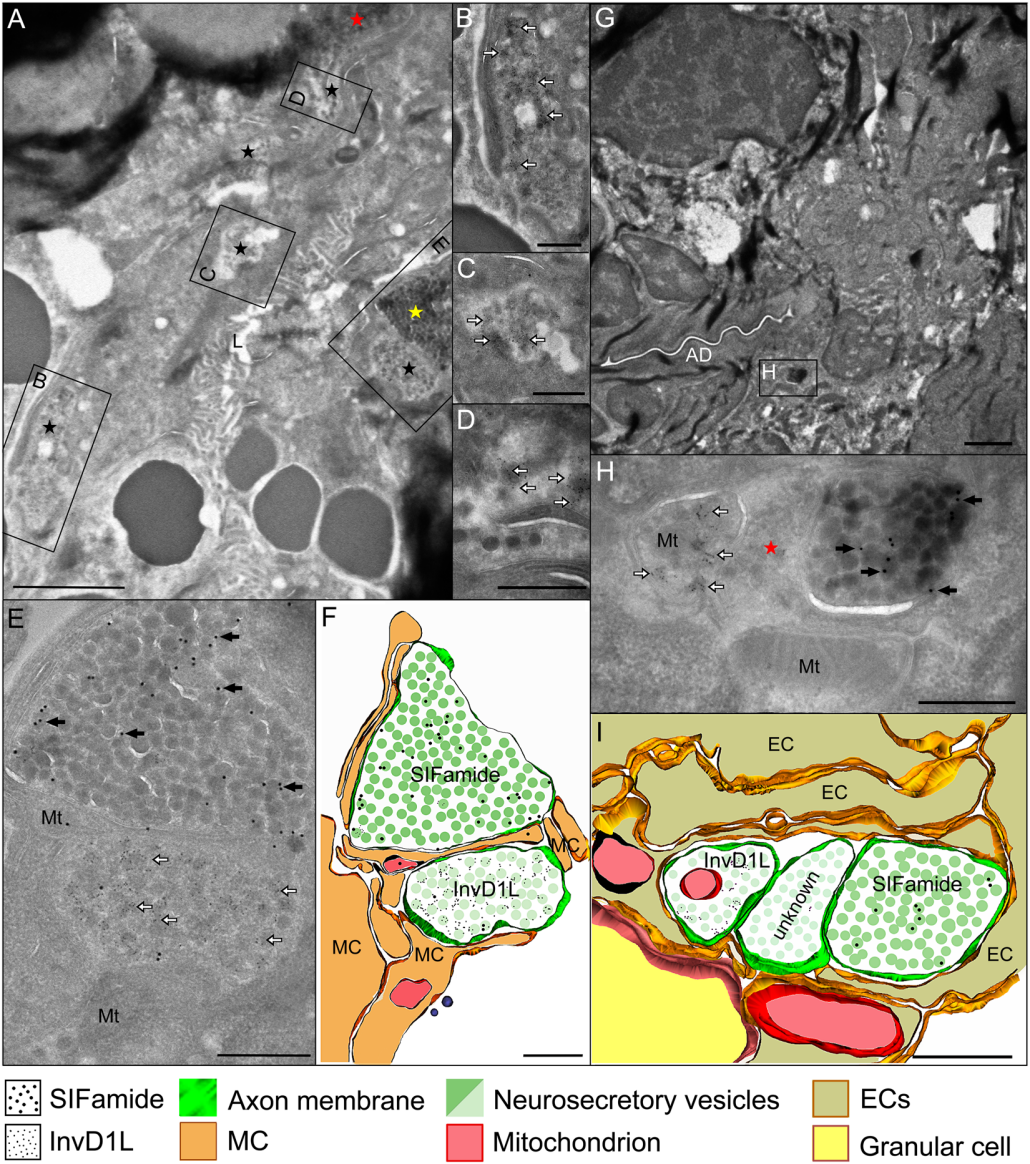
Transmission electron microscopy image showing double immunogold labeling of SIFamide and invertebrate-specific D1-like dopamine receptor (InvD1L) in both type II (**A–F**) and III (**G–I**) acini of unfed *Ixodes ricinus* female salivary glands. (**A**) Cross section of type II acinus highlighting the axons running close to the acinar lumen (L). Five InvD1L-axons (black asterisk) containing electron-lucent vesicles, one SIFa-axon (yellow asterisk) containing electron-dense vesicles and one unlabeled axon (red asterisk) containing electron-dense vesicles were recognized. Insets in (**A**) are magnified in (**B–E**). InvD1L- (white empty arrows show the 6 nm nanoparticles in (**B–E**) and SIFamide- (black arrows show 15 nm nanoparticles in **E**) reactions are shown. (**F**) 3D model of (**E**) emphasizing the axons and surrounding MC. Numerous mitochondria (Mt, red in **F**) were detected in the MC surrounding these axons. (**G**) Basal region of type III acinus highlighting three different axons running alongside close to the acinar duct (AD). Inset in (**G**) is magnified in (**H**). InvD1L-axons (white empty arrows show the 6 nm nanoparticles in **H**) containing electron-lucent vesicles, SIFa-axon (black arrows show 15 nm nanoparticles in **H**) containing electron-dense vesicles and an unlabeled axon (red asterisk) containing electron-lucent vesicles were detected. (**I**) 3D model of (**H**) emphasizing the axons and surrounding basal epithelial cells (ECs). Numerous mitochondria (Mt, red in **I**) were detected in the ECs and the InvD1L-axon. Scale bars: 2 μm (**A**,**G**), 500 nm (**B–F,H,I**).

**Figure 7:**
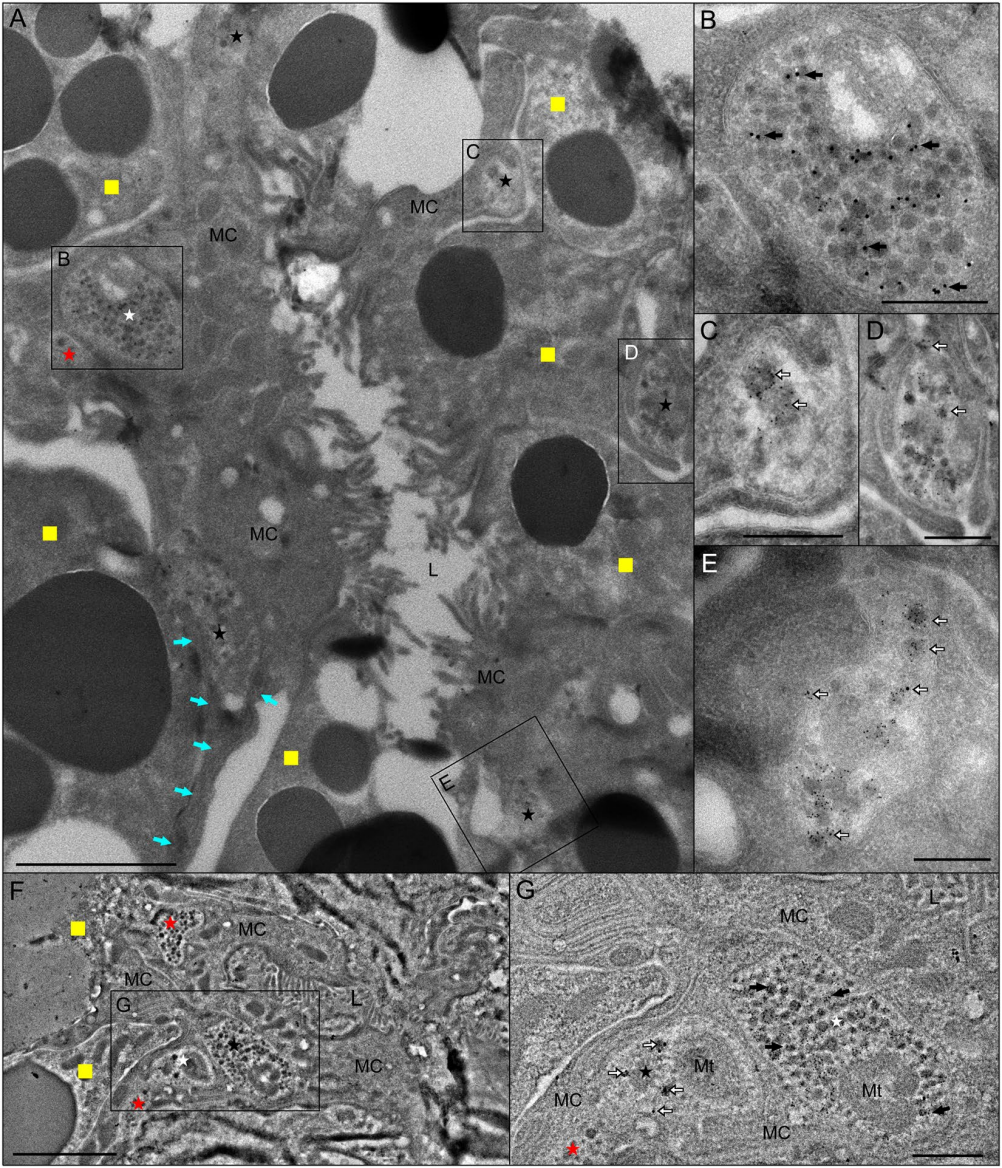
Transmission electron microscopy image showing double immunogold labeling of pigment dispersing factor (PDF) and invertebrate-specific D1-like dopamine receptor (InvD1L) in type II acini of unfed *Ixodes ricinus* female salivary glands. (**A**) Cross section of type II acinus detailing the axonal projections arranged around the acinar lumen (L). Insets in A are magnified in (**B–E)**. The PDF-axon (white asterisk) containing large electron-dense vesicles was visualized using 15 nm nanoparticles (black arrows in **B**), whereas InvD1L-axons (black asterisks) containing electron-lucent vesicles were visualized with 6 nm nanoparticles (empty white arrows, in **C–E**). An unlabeled axon running close to the PDF-axon (red asterisk in **A**) containing electron-dense vesicles was also observed. Note that the axons were primarily encapsulated with the plasma membrane of the myoepithelial cell (MC) and terminal projections of ablumenal interstitial cells (AEC, aqua-blue arrows) while less frequent connections with granular cells (yellow squares) were also observed. (**F**,**G**) Another region of type II acinus shows three distinct axons running alongside and surrounded by the MC plasma membrane. Inset in F is magnified in (**G)**. A bundle of three axonal processes (InvD1L- 15 nm nanoparticles black arrows; PDF-6 nm nanoparticles white arrows, unlabeled axon, presumed to express SIFamide, is showed by red asterisks) enclosed by the MC in the area of the lumen. Mitochondria (Mt) were evident in both PDF- and InvD1L-axons (**G**). Bars: 2 μm (**A,F**), 500 nm (**B–E,G**).

The InvD1L-positive axons (InvD1L-axons) containing electron-lucent neurosecretory vesicles were the most abundant axons in both type II and III acini (Figs. 6 and 7). Double staining of InvD1L and SIFamide in type II acini distinguished seven axonal projections scattered around the acinar lumen: five InvD1L-axons, one SIFa-axon, and one axon that was negative for both antibodies (Fig. 6A–F). The InvD1L- and the SIFa-axons often ran alongside each other (Fig. 6E,F), while solitary InvD1L-labeled axons (Fig. 6B,C), or InvD1L-axons (Fig. 6A,D) associated with unlabeled axons were also observed. InvD1L-axons appeared to have smaller diameter (~1–2 μm) along their whole length than SIFa-axons (~2–5 μm) (Fig. 6E,F). All detected axons were predominantly enclosed by the ablumenal plasma membrane of MC, and frequent contacts with the fine AEC projections were also identified. No axons were observed to directly contact the acinar lumen.

In the basal region of type III acini, three distinct axons running in parallel along the acinar duct were identified: an InvD1L-axon, a non-labeled axon, and an SIFa-axon (Fig. 6G–I). In this region all three axons ran in close contact, and were enclosed by convoluted EC plasma membranes. Axons differed in the electron density of their secretory vesicles; both the unlabeled and the InvD1L-axon were electron-lucent, whereas the SIFa-axon was electron-dense. Surrounding ECs contained multiple mitochondria while these organelles were also observed in the InvD1L-axon (Fig. 6H,I).

### Pigment dispersing factor and invertebrate-specific D1-like dopamine receptor

The antibody against PDF recognized distinct axonal projections adjoining the acinar lumen exclusively in type II acini (Fig. 7). Double labeling of PDF and InvD1L distinguished seven axonal projections on the cross section of the acinus: a single large-caliber (~2 μm) PDF-positive axon (PDF-axon) containing electron-dense vesicles (Fig. 7B), five proportionally smaller diameter (~1 μm) InvD1L-axons containing electron-lucent vesicles (Fig. C–E) and one axon that was negative for both PDF and InvD1L and which contained electron-dense vesicles, presumed to be the SIF-axon (Fig. 7B,F,G). The axons often ran alongside each other in the basal region of the acinus (Fig. 7F,G), whereas more distant separation was observed in the region surrounding the acinar lumen (Fig. 7A). Serial TEM sectioning revealed that PDF-axons, along with the other axons surrounding the acinar lumen, were primarily enclosed by the ablumenal plasma membrane of MC (Fig. 7A,F), and that direct contact with AEC terminal extensions was also detected.

### Results summary

**Figure 8:**
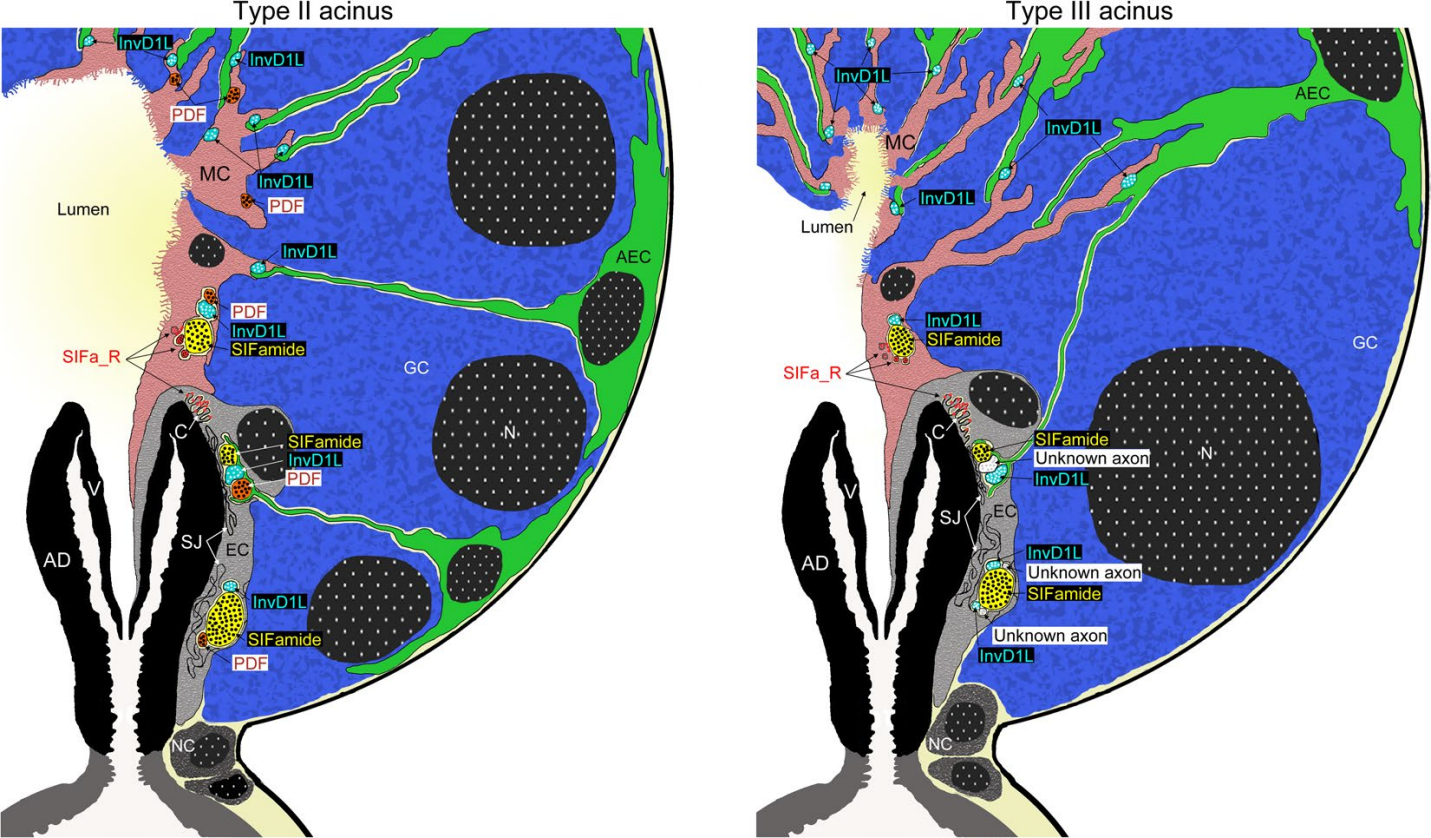
Schematic illustration demonstrating cross sections of type II and III acini from unfed *Ixodes ricinus* female salivary glands highlighting the organization of acinar cells and their putative associations with different axonal projections. SIFamide-positive axons (yellow), pigment dispersing factor-positive axons (PDF, brown/orange), invertebrate-specific D1-like dopamine receptor-positive axons (InvD1L, aqua-blue). SIFamide receptor (SIFa_R, red): on plasma membrane and vesicle-like structure (upper arrow) of the myoepithelial cell (MC, pink), axons accompanying the SIFa-axon surrounded by the MC (middle arrow) and canaliculi (lower arrow) of basal epithelial cell (EC, grey). Note that acinar valve (V) is covered by EC extensions and overlaid by the MC. AEC, ablumenal epithelial cell: green; GC, granular cell: blue; NC, neck cells: dark grey; N, nucleus: dotted black; AD: acinar duct; C: canaliculi of ECs; SJ: septate junctions. For description of different granular cell types in hard tick SG see^4,5^.

The current study reveals several novel ultrastructural architectural features of SG type II and III acini from unfed *I. ricinus* females. Significant attention has been paid to specific axonal projections and their associations with acinar cells (Fig. 8).

- Both type II and III acini comprise various combinations of four common cell types able to form highly complex secretory units. (1) ECs closely surround the acinar duct and its valve. (2) A single mesh-like MC lines the entire acinar lumen and extends its finger-like projections between granular cells toward the basement membrane. (3) Various granular cell types surround the acinar lumen and make direct contact with it via the mesh-like openings in the MC. (4) AEC interstitial extensions radiate between the granular cells toward the acinar lumen to make contact with the MC and/or ECs. AECs are never in direct contact with the acinar lumen. The MC is in contact with all acinar cells.
- The acinar valve is covered by two layers of cells: apical EC extensions line the entire length of the valvular arms, while the MC cell overlays nearly half of these extensions. Both the MC and ECs possess abundant microtubules, primarily located near the acinar valve.
- All axonal projections enter the acini via their neck regions and run adjacent to the acinar lumen. At the very base of the acini, axons are encapsulated by convoluted plasma membranes of interdigitated ECs often specialized in septate junctions. Axons located closer to the apex of the acinar duct are enclosed by ECs and fine AEC terminal extensions. In the apical acinar region, axons are enclosed by the MC and terminal AEC extensions. The axons may come into occasional contact with acinar granular cells.
- SIFamide and SIFa_R are associated with both ECs and the MC. Large SIFa-axons are encapsulated by EC plasma membranes around the acinar duct or the MC cell adjoining the region above the acinar valve. Accordingly, SIFa_R reactions were also detected, always in close proximity to SIFa-axons. Specifically, SIFa_R is expressed on EC canaliculi and the MC plasma membrane, both near the apex of the acinar duct and its valve. In addition, SIFa-R-positive axons surrounding the SIFa-axon are encapsulated by the MC plasma membrane.
- InvD1L-axons run the entire length of the acinus, in close proximity to its lumen. These axons are primarily enclosed by the ablumenal membrane of MC and terminal AEC extensions. These axons are occasionally in contact with apical granular cells.
- PDF-axons surround the lumen of type II acini only. These axons reach the apical region of the acini, and are in direct contact with the MC plasma membrane and terminal AEC extensions.
- In type III acini, the axon expressing unknown substance(s) and containing electron-lucent vesicles runs along the SIFa- and InvD1L-axons, and is likely only specific to this type of acini.

## Discussion

Numerous earlier studies have described the presence of axonal projections in hard tick SG^11,15–19,21,24^ and it has since been suggested that neuronal commands are the primary regulatory factors involved in tick salivary secretions^10,14,23,25^. Over the last decade, the use of specific antibodies in wholemount immunohistochemistry has led to the identification of several different specific innervations expressing either neuropeptides or neurotransmitter receptors in hard tick SGs^16,18–22^. Our in-depth ultrastructural investigations have clarified previous findings and have also enabled the generation of new insights about the organization of SG acinar cells in *I. ricinus*, the primary Lyme disease vector in Europe^29^.

Although the ultrastructure of hard ticks SGs has been extensively studied^11–13,24,30,31^, very little is understood about the distribution of axonal processes in specific acini types. In our study, all axonal projections of type II and III acini enter via acini neck regions and run together along acinar ducts toward the apex. In this region, apparent varicosities of some of the axons (see below) suggest a presynaptic-release of signaling molecules to the surrounding target effector cells, while other axons may use this zone exclusively as a passageway to the apical acinar regions. Basal ECs have been found to be rich in mitochondria, indicating an active energy supply for transcellular transport in this region. This assumption is also supported by the presence of numerous septate junctions between ECs, probably regulating paracellular transport across the epithelial layers. The close proximity of septate junctions to basal axons indicate that these structures may be targets for the axonal products, but more in-depth investigation is necessary to confirm this assumption. In invertebrates, numerous different functions have been recently ascribed to these highly specialized membrane domains^32^, and although septate junctions have been observed in SG of various tick species^13,31^, their molecular organization in this tissue requires further examination. The use of hafnium staining instead of conventional uranyl acetate in our experiment greatly enhanced EC and AEC cytoplasmic contrast. We hypothesize here that improved hafnium staining of EC—and possibly AEC—cytoplasm is caused by ionic diffusion facilitated by the presence of septate junctions between ECs. This assumption is supported by previous reports that invertebrate septate junctions serve as passages for different ionic probes^33^ and in ticks nervous system these structures have been shown freely permeable to lanthanum electron-dense tracer^34^.

A single adlumenal MC, also called a cap cell, has been identified in previous reports^12,19,24^, but the intact nature of this cell has now been shown for the first time in this study via TEM sections. The spatial organization of this cell in combination with abundant cytoplasmic microtubules, supports a role whereby the MC mediates acini contractions, thus expulsing their contents into the adjacent ducts^19,23,25^. In addition, we reveal here that the basal portion of the MC covers the acinar valve arms, again supporting our prior hypothesis^19,23,25^ of MC-mediated valve control. Based on our observations, the interconnected MC is in contact with all acinar cells as well as all axonal projections within the acinus. We showed that apical MC projections along AEC terminals often enclose axons to form MC-axon-AEC features. Similarly, ECs along AEC terminals enclose axon clusters near the acinar duct. These observations indicate that a common neural mechanism controls two different cell types by one or more axonal projections, but at this point, further studies are essential to fully understand the physiological role(s) of their synaptic interactions. In a few of our TEM slices, we occasionally observed axon connections with granular cells. These connections were the exception rather than the rule, and were only noted at locations were axons were already in contact with either ECs/AECs, MC/AECs or the MC. Based on this observation, it appears that different axons may selectively control ECs, AECs, and the MC (see below), while direct neuronal commands for granular cell activities need to be further investigated.

The large-caliber SIFa-axons containing electron-dense vesicles were the most prominent axons in the acinar basal region in both acini types II and III. These axons are typical by the large varicosities abundant along the whole length of their terminals in tick SG^21^. Large electron-dense axons, neighboring the acinar duct, were identified in previous ultrastructural studies^11,13^ and along with the SIFa-axons identified in numerous hard tick species^17,21,22^, suggest a common neuropeptidergic control of the SG in the hard tick lineage. Wholemount IHC of acini type II and III in our previous report showed SIFa_R expression at both the region surrounding the acinar duct and on apically-spreading fiber-like structures on the lumenal acini surface^20^. Immunogold staining of SIFa_R in the current study appears to confirm previous findings, however it has also generated a much more detailed picture of the receptor’s spatial expression in the basal region of the acini. Specifically, SIFa_R expression was detected at the basal canaliculi of ECs (in both acini types), as well as on the MC plasma membrane (in type III acini), both in close proximity to the acinar valve. Here the SIFa_R-positive vesicle-like structures observed in MC may belong to the Golgi apparatus or the endoplasmic reticulum, where synthesized receptor is being transported to the MC plasma membrane. Considering that both ECs and the MC overlay the valvular arms, we propose that SIFamide/SIFa_R play a joint role in regulating these structures by activating downstream effector proteins in these two cell types. At this point it is difficult to predict whether ECs and MC control the acinar valve in an agonistic or antagonistic fashion, but is reasonable to believe that their downstream cellular responses are regulated by different antagonistic/agonistic neuropeptides. This assumption is supported by the fact that in addition to SIFamide, these axons are also known to co-express MIP and elevenin neuropeptides in both type II and III acini^18,21^, although the ultrastructural receptor localization remains to be established. Furthermore, identifying fine SIFa_R-positive axons surrounding the prominent SIFa-axon is an interesting finding suggesting another physiological role for SIFamide in tick SGs. The SIFa_R-positive axon, containing electron-lucent vesicles, likely corresponds to the fiber-like structures previously identified with IHC^20^. Here, we speculate that electron-lucent InvD1L-axons, often found to run alongside SIFa-axons, may also co-express SIFa_R, although double staining of InvD1L and SIFa_R is not currently possible due to the antibodies’ common host species. Thus, in addition to controlling acinar valve activities, SIFamide may also play a role in the neuromodulation of adjoining axonal projections to inhibit or facilitate the release of unknown neurotransmitter(s).

In our earlier wholemount IHC of *I. scapularis* SG, the InvD1L receptor antibody recognized specific axonal processes but also cross-reacted with SIFa-axons in both type II and III acini^19^. Interestingly, double immunogold labeling with anti-SIFamide and -InvD1L dopamine receptor antibodies in the current study recognized individual axons exclusively expressing either SIFamide or InvD1L dopamine receptor, in both acini types. These discrepancies could be explained by the different epitopes retrieved by these two techniques, which may lead to non-specific binding of InvD1L receptor antibody to an unknown protein in SIFa-axons. InvD1L-axons were the most abundant in both type II and III acini and their termini reached the most apical acinar regions. Direct associations of InvD1L-axons with the numerous MC zones support a proposed model where InvD1L activation triggers Ca^++^-mediated MC contraction and expulsion of acinar contents^19,23,25^. In addition to the MC, for which it is always the case, we found that InvD1L-axons frequently contacted AEC projections and also occasionally granular cells. During tick feeding, the AECs, also called “water cells” in earlier studies^4,8^, and granular cells undergo remarkable transformation^4^, likely as a direct response to the acute needs of massive saliva production in order to meet the challenge of the host defense systems^2,3^. As a result of this process, the type II and III acini dramatically increase their overall volume and form a specialized glandular epithelium for active fluid transport from the surrounding haemolymph^4,8,31^. Considering that InvD1L axons are in intimate contact with the majority—if not all—acinar cells, we speculate that in addition to directly controlling MC contractions, InvD1L-axons may also regulate SG acini development during feeding via an unknown neurotransmitter/neuromodulator. This assumption is supported by the complete absence of immunoreactive InvD1L-axons during tick feeding in type III acini^19^, that are known to undergo more dramatic transformations than type II acini^30^.

The presence of PDF-axons found exclusively in type II acini confirmed previous IHC findings^16,17,22^. Similarly to InvD1L-axons, we observed that PDF-axons directly contacted the MC and AEC terminal extensions. We speculate that in type II acini, PDF-axons mediate the activity of the MC, and possibly AECs, to ensure the temporal secretory needs of type II acini during tick feeding. The PDF gene has not yet been identified in any tick species, thus further studies are required to identify the specific neuropeptides being released from these axonal projections. In addition, the anti-orcokinin antibody, known to recognize PDF-axons in wholemount IHC approaches^22^, failed in our TEM staining. To date, there has been no evidence that the axonal projections specifically and exclusively target type III acini, thus SIFa/MIP/Elv- and IvD1L-axons remain the only candidates for neural control of these structures^18,19,21^. Interestingly, in the current study, double staining with SIFamide and InvD1L antibodies uncovered an additional electron-lucent axon that failed to react with either of these antibodies, indicating a novel axonal projection only identified in type III acini. This finding, along with the identification of type II acini-specific PDF-axons, indicates that ticks could selectively regulate the activities of specific type of acini in direct response to their physiological needs.

In the present study, we have described for the first time the ultrastructure of specific axonal processes reaching type II and III SG acini in *I. ricinus*, a major European disease vector. Our results indicate that a rich network of various axon types, differing in their lengths, thicknesses, and expressed substances, form intimate contacts with multiple different acinar cell types, highlighting the complexity of the neural regulatory mechanisms in hard tick SGs.

## Supplementary information

Supplementary information

Video 2

Video 1

## Data Availability

All data generated or analyzed during this study are included in this published article (and its Supplementary Information files).
