# Supplementary material for: Ultrastructural mapping of salivary gland innervation in the tick Ixodes ricinus
Source: Sci Rep. 2019 May 2;9:6860. doi: 10.1038/s41598-019-43284-6 (PMC6497691; doi:10.1038/s41598-019-43284-6)
Supplement: Supplementary file 1 — Supplementary information [file 41598_2019_43284_MOESM1_ESM.doc]

**Supplementary information**

**Ultrastructural mapping of salivary gland innervation in the tick *Ixodes ricinus***

Marie Vancová^1,2^, Tomáš Bílý^1,2^, Jana Nebesářová^1,3^, Libor Grubhoffer^1,2^, Sarah Bonnet^4^, Yoonseong Park^5^ and Ladislav Šimo^4*^

^1^Laboratory of EM, Institute of Parasitology, Biology Centre of CAS, České Budějovice, Czech Republic,

^2^Faculty of Science, University of South Bohemia, České Budějovice, Czech Republic,

^3^Faculty of Science, Charles University in Prague, Czech Republic

^4^UMR BIPAR, INRA, Ecole Nationale Vétérinaire d’Alfort, ANSES, Université Paris-Est, Maisons-Alfort, France

^5^Department of Entomology, Kansas State University, 123 Waters Hall, Manhattan, KS 66506, USA

*Corresponding author: [ladislav.simo@vet-alfort.fr](mailto:ladislav.simo@vet-alfort.fr)

*I. ricinus* SIFa_R ..IKSRSCCGTLREPSYSVRGTTLRSGN**TRGLSRYDTQCEYLSTSAV***

*I. scapularis* SIFa_R ..IKSRSCCGTLREPSYSVRGTTLRSGN**TRGLSRYDTQCEYLSTSAV***

*I. ricinus* IvD1L ..IYACWSRDFRRAFANVLCCCCPGYFRRRQRRRDRL**RRIIKEDASMRSQSLEEAVL***

*I. scapularis* IvD1L ..IYACWSRDFRRAFANVLCCCCPGYFRRRQRRRDRL**RRIIKEDASMRSQSLEEAVL***

**Supplementary Figure S1.** Alignment of the N-terminal sequences of SIFamide receptor (SIFa_R) and invertebrate specific D1-like (InvD1L) dopamine of *Ixodes ricinus* and *Ixodes scapularis.* The immunoreactive epitopes are highlighted in yellow. Note that the immunoreactive epitopes (yellow) share the 100% identity.


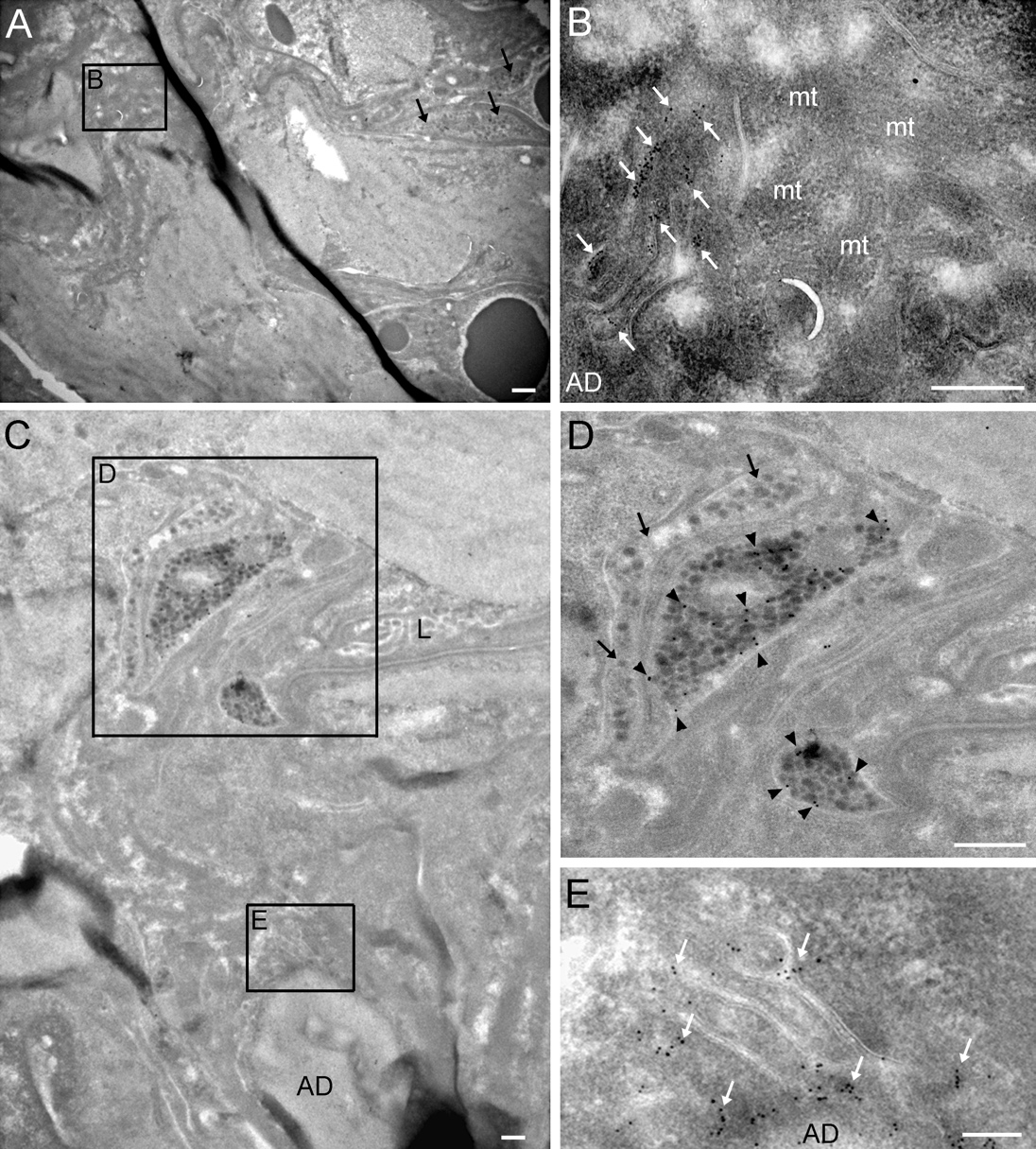


**Supplementary Figure S2.** Transmission electron microscopy image showing immunogold labeling of SIFamide and SIFamide receptor (SIF_R) in type II acinus of unfed *Ixodes ricinus* female salivary glands. (A) Basal area of the acinus type II. Axons with electro-lucent vesicles, presumed to be InvD1L-positive are shown by black arrows. (B) Magnified region of image A, where SIFa_R staining (white arrows show 6 nm nanoparticles) was found on the EC canaliculi associated with the acinar duct (AD) apex region. Microtubules (mt). (C) Double staining of type II acinus basal region with the SIFamide and SIFa_R. Insets in C are magnified in D and E. Note that SIFa-positive axons (15nm nanoparticles - black arrowheads) are accompanied by axons with electro-lucent granules (presumably IvD1L axons – black arrows). SIFa_R-positive reaction (6nm nanoparticles – white arrows) is shown on the EC canaliculi, associated with the acinar duct (AD) apex region. L – lumen. Bars 500 nm.

**Video Legends**

**Video 1.** 3D reconstruction of TEM sections (Fig. 3A) highlighting the association of basal epithelial cells (ECs) and ablumenal interstitial cells (AECs) with axonal projections running along the salivary duct. Note that axons running along the salivary duct are tightly encapsulated by ECs cells and terminal extensions of AECs forming ECs-axon-AECs features. Occasional contact of axons with secretory cell was also observed. Green: acinar duct/valve; blue: ECs; red: axons; aqua blue: AECs; orange/yellow: cell nuclei, grey: secretory cell.

**Video 2.** 3D reconstruction of TEM sections (Fig. 5B) highlighting SIFamide-positive axon (SIFa-axon) with associated structures near the acinar lumen. Note that large caliber SIFa-axon is surrounded by four smaller axons negative to SIFamide staining. All axons are enclosed by convoluted plasma membrane of ECs. Septate junctions between ECs are apparent between acinar duct and the axons. For better understanding of examined structures, showed in different colors, see Figure 5.
